# DNA extraction methods and pre-analytical workflows for next-generation sequencing in infected dental pulp and endodontic infections: A scoping review

**DOI:** 10.4317/jced.64251

**Published:** 2026-09-15

**Authors:** María Mihaela Iuga, Tatiana Ramírez-Mora, Bogdan Oprea, Fernando Mauricio Espada-Salgado

**Affiliations:** 1 Faculty of Health Sciences, Jorge Basadre Grohmann National University, Tacna, Perú; 2 Restorative Department, Faculty of Dentistry, University of Costa Rica, San José, Costa Rica; 3 Department of Dental Morphology, University Lucian Blaga Sibiu, Sibiu, Romania; 4 Faculty of Health Sciences, Private University of Tacna, Tacna, Perú

**Keywords:** DNA extraction, endodontics, infected dental pulp, next-generation sequencing, pre-analytical workflow, root canal infections.

## Abstract

**Background:**

Next-generation sequencing (NGS) has improved the characterization of microbial communities in infected dental pulp and endodontic infections. However, variability in sampling procedures, DNA extraction methods, contamination control, and quality assessment may affect microbial recovery and limit comparability across studies. This scoping review mapped the available evidence on DNA extraction methods and pre-analytical workflows used for NGS in human infected dental pulp and endodontic infections.

**Materials and Methods:**

This scoping review was conducted according to Joanna Briggs Institute guidance and reported following PRISMA-ScR. Searches were performed in Scopus, Web of Science Core Collection, Embase, PubMed/MEDLINE, and LILACS, with additional screening of Google Scholar and ClinicalTrials.gov, from 2011 to March 23, 2026. Records were deduplicated in Zotero and screened in Rayyan by two independent reviewers, with disagreements resolved by discussion or a third reviewer. Data were descriptively charted for specimen type, sampling procedures, storage, contamination control, lysis and extraction methods, sequencing platforms, bioinformatic processing, and quality metrics.

**Results:**

Of 389 records identified, 219 remained after duplicate removal, 44 full-text articles were assessed, and 30 studies were included. Most studies used short-read 16S amplicon sequencing, mainly on Illumina platforms. Specimens included paper-point samples, endodontic files, pulp tissue, cryopulverized root segments, root apices, and periapical lesions. Substantial heterogeneity was observed in lysis strategies, extraction kits, matrix processing, contamination controls, and pre-sequencing quality assessment.

**Conclusions:**

Pre-analytical heterogeneity limits reproducibility and cross-study comparability in endodontic NGS research. Standardized reporting, matrix-specific DNA extraction validation, systematic contamination controls, and transparent quality metrics are needed to strengthen future endodontic microbiome studies.

## Introduction

Endodontic infections are polymicrobial diseases in which microbial communities within the root canal system play a central role in the development and persistence of apical periodontitis. Next-generation sequencing (NGS) has expanded the understanding of these infections by showing broader and more heterogeneous microbiota than those detected by culture-based or targeted molecular methods. A recent systematic review highlighted an important limitation in this field: Substantial methodological variability across studies, especially in sample collection and microbial analysis [1].

Recent human studies show that the microbiome of endodontic infections varies according to infection type, anatomical site and treatment status. Comparative NGS studies have reported differences between primary and persistent or secondary infections [2], whereas paired analyses of root apices and periapical lesions have shown that microbial composition may also be associated with clinical presentation [3]. In post-treatment disease, candidate keystone taxa have been identified [4], and studies in primary apical periodontitis have confirmed that chemo mechanical preparation can significantly alter the intracanal bacteriome without eliminating its complexity [5,6].

Sequencing results are only as reliable as the workflow used before sequencing begins. In endodontic microbiome research, the pre-analytical phase includes field disinfection, sampling method, transport and storage conditions, lysis procedures, DNA extraction, contamination control and nucleic acid quality assessment. This is especially relevant in endodontics, where samples may contain low microbial biomass, abundant host material and a high risk of procedural contamination. Recent methodological studies have shown that different *in vivo* sampling strategies can produce different microbial profiles [7], and that infected dental pulp may require tissue-specific extraction protocols to obtain DNA of sufficient quality for downstream NGS analysis [8]. More recent metagenomic work in irreversible pulpitis further supports the need for robust and standardized workflows [9].

Taken together, the available evidence indicates that NGS is increasingly relevant in endodontic research, but methodological inconsistency still limits reproducibility and meaningful comparison across studies. Therefore, a focused synthesis of pre-analytical workflows and DNA extraction methods used for NGS in human endodontic and pulpal infections is needed to guide future study design and strengthen the clinical relevance of sequencing-based findings.

## Materials and Methods

*Design and reporting*

This scoping review was conducted in accordance with the methodological guidance proposed by the Joanna Briggs Institute for scoping reviews and reported following the Preferred Reporting Items for Systematic Reviews and Meta-Analyses extension for Scoping Reviews (PRISMA-ScR) [10,11]. An a priori protocol and supporting review materials were prospectively deposited in the Open Science Framework (OSF) before completion of the review process and are available at: https://osf.io/f5eq6/.

*Review question and eligibility criteria*

The review question was structured according to the Population-Concept-Context (PCC) framework. The primary review question was: In human endodontic infection contexts, what pre-analytical and DNA extraction workflows have been used for next-generation sequencing-based microbial profiling, and what operational steps and performance or quality metrics are reported?

The population comprised human endodontic samples obtained from teeth with infected dental pulp, pulpal necrosis and/or endodontic infections, including dental pulp, root canal contents, intraradicular dentin or biofilm scrapings, root canal exudates, paper points, files or instruments carrying canal material, and periapical samples clearly associated with an endodontic infection, such as apical periodontitis or periapical lesions of endodontic origin.

The concept of interest was the set of pre-analytical and DNA extraction workflows used for NGS-based microbial profiling. These included sampling and handling procedures, asepsis or isolation protocols, contamination prevention, transport and storage conditions, sample preservation, pre-treatment and lysis strategies, DNA extraction and purification methods, inhibitor removal, host DNA depletion or microbial enrichment procedures when applicable, and reported quality-control or performance metrics, such as DNA yield, purity, integrity, inhibition assessment, contamination controls, and sequencing output metrics.

The context included clinical, laboratory, or *ex vivo *studies using human teeth or human endodontic samples, conducted in any country or setting, without language restrictions, from 2011 onward.

Studies were eligible if they included human endodontic or pulp-related samples from infected pulp, pulpal necrosis and/or endodontic infections; used NGS approaches such as 16S amplicon sequencing, shotgun metagenomics, or metatranscriptomics, or explicitly evaluated or validated a pre-analytical or DNA extraction workflow intended for NGS applications; and reported at least one relevant operational component, such as sample preservation or transport, lysis strategy, DNA extraction or purification method or kit, contamination controls, DNA quality or performance metrics, or sequencing output metrics. Eligible study designs included methodological kit or protocol comparisons, technical validations, clinical or observational studies, case series reporting the workflow, and *ex vivo* studies using human teeth when clinically relevant.

Studies were excluded if they did not use NGS, such as culture-only, standalone PCR/qPCR, or Sanger-only studies; were not related to endodontic or pulpal infection contexts; focused exclusively on saliva, supragingival or subgingival plaque, periodontal microbiome, gingivitis, or caries unrelated to endodontic infection; were based exclusively on pure isolates without DNA extraction from an endodontic or pulpal clinical sample as the primary source; lacked sufficient methodological detail for workflow mapping; used animal models; or were editorials, commentaries, opinion papers, letters, narrative reviews, or conference abstracts without adequate methodological reporting.

*Search strategy and study selection*

A comprehensive search strategy was developed and adapted for each information source. The electronic databases searched were Scopus (Elsevier, Amsterdam, the Netherlands), Web of Science Core Collection (Clarivate, London, United Kingdom), Embase (Elsevier, Amsterdam, the Netherlands), PubMed/MEDLINE (National Library of Medicine, Bethesda, MD, USA), and LILACS through the Virtual Health Library (Latin American and Caribbean Center on Health Sciences Information, São Paulo, Brazil). Grey literature and ongoing or registered studies were additionally explored through Google Scholar (Google LLC, Mountain View, CA, USA) and ClinicalTrials.gov (U.S. National Library of Medicine, Bethesda, MD, USA), respectively. The search strategy combined controlled vocabulary and free-text terms related to next-generation sequencing, DNA extraction, pre-analytical processing, and endodontic or pulpal infections, while excluding studies focused on saliva, dental plaque, periodontal conditions, gingivitis, and caries. The final search was conducted on March 23, 2026. The complete search strategies for each database, grey-literature source, and trial register are provided in Supplementary Table S1 (https://www.medicinaoral.com/medoralfree01/64251/Suppl1.pdf).

All retrieved records were imported into Zotero 7 (Corporation for Digital Scholarship, Vienna, VA, USA), where duplicate citations were identified and removed before screening. The deduplicated records were subsequently exported to Rayyan, a web-based systematic review platform (Rayyan Systems Inc., Cambridge, MA, USA; accessed March 2026), to support the study-selection process [12]. Screening was conducted in two stages: Title and abstract screening, followed by full-text assessment according to predefined eligibility criteria. Two reviewers independently screened the records at both stages. Disagreements were resolved through discussion; when consensus could not be reached, a third reviewer acted as an adjudicator. A detailed full-text screening table, including the final inclusion and exclusion decisions and the reasons for exclusion, is provided in Supplementary Table S2 (https://www.medicinaoral.com/medoralfree01/64251/Suppl2.pdf).

*Data charting and synthesis*

Data were charted using a structured extraction form developed specifically for this review. Extracted information included bibliographic characteristics, country and setting, study design, infection or sample type, sampling and aseptic procedures, transport and preservation methods, pre-treatment or lysis strategy, DNA extraction or purification method or kit, inhibitor removal, host DNA depletion or microbial enrichment procedures when applicable, contamination controls, DNA yield, purity, integrity or other performance metrics, sequencing approach or platform, bioinformatic processing, reference database, and main workflow-related findings.

For evidence mapping, contamination control was classified as reported only when the full text explicitly described a sampling or operative-field control, procedural blank, extraction blank, no-template amplification control, reagent or library control, mock control, or another specific contamination-monitoring sample. Preventive aseptic procedures without a corresponding control sample or blank were not classified as contamination controls.

As recommended for scoping reviews, no formal critical appraisal of the included studies was performed because the aim was to map methodological evidence rather than to assess effect estimates, diagnostic accuracy, or intervention efficacy [10,11]. The findings were synthesized descriptively and presented in tables, figures, and narrative form, emphasizing recurrent methodological steps, matrix-specific workflow characteristics, sources of heterogeneity, quality-control practices, and reporting gaps relevant to reproducibility in endodontic NGS research. Descriptive counts and figures were generated using Python 3.12.13 (Python Software Foundation, Wilmington, DE, USA) and Matplotlib 3.10.8 (Matplotlib Development Team, USA).

## Results

*Search results and study selection*

The database and grey-literature searches identified 389 records: Scopus (n=91), Web of Science Core Collection (n=50), PubMed/MEDLINE (n=26), Embase (n=121), LILACS (n=4), Google Scholar (n=96), and ClinicalTrials.gov (n=1). After the removal of 170 duplicate records, 219 records were screened by title and abstract. Of these, 175 were excluded, and 44 reports were sought for retrieval and assessed for full-text eligibility. Thirty studies met the eligibility criteria and were included in the review. Fourteen full-text reports were excluded for the following reasons: Absence of an NGS-based approach (n=9), insufficient reporting of the pre-analytical or DNA extraction workflow (n=2), population or context not aligned with the PCC framework (n=2), and study protocol without primary results (n=1) (Figure 1).

*Characteristics of the included studies*

The 30 included studies were published between 2011 and March 23, 2026. The highest annual number of included studies was observed in 2024 (n=5), followed by 2018, 2022, and 2025 (n=4 each). Because the literature search covered only the first part of 2026, the number of studies identified for that year should be interpreted as incomplete (Figure 2).

Twenty-eight studies generated NGS datasets from human clinical or surgical samples, whereas two were methodological optimization or validation studies focused on improving DNA isolation from infected dental pulp or dental hard tissue without generating sequencing datasets [2-9,13-34]. Root canal or intraradicular sampling was the most frequent specimen type (14/30), followed by apical root or cryopulverized hard-tissue specimens (6/30), pulp tissue (4/30), and paired root apex-periapical tissue samples (3/30). The remaining studies used paired or adjunct extraradicular sampling designs, including root canal samples combined with extraradicular or periapical material or gingival sulcus samples.

Short-read 16S rRNA gene amplicon sequencing predominated (26/30 studies). One study used full-length 16S rRNA gene sequencing with Oxford Nanopore technology, one used shotgun metagenomic sequencing, and two focused exclusively on DNA-extraction optimization or validation without generating sequencing datasets. Among the 28 studies that generated NGS datasets, Illumina MiSeq was the most frequently used platform (17/28), followed by 454 pyrosequencing (6/28), Illumina HiSeq (2/28), Illumina NovaSeq (2/28), and Oxford Nanopore technology (1/28) (Table 1, Figure 3).

*Sample acquisition and pre-analytical handling*

Reporting of sample acquisition and pre-analytical handling was heterogeneous. Among the 30 included studies, 29 described an aseptic operative, surgical, or sample-retrieval procedure, whereas asepsis was not applicable to one laboratory-based methodological study. The reported procedures commonly included rubber dam isolation, disinfection of the operative field or tooth surface, antisepsis of the surgical site, and the use of sterile sampling instruments. Intraradicular samples were usually collected using sterile paper points, endodontic files, or a combination of both. Studies evaluating apical or hard-tissue specimens generally used surgically obtained root tips that were subsequently cryoground, cryopulverized, or mechanically ground. Pulp-focused studies collected inflamed pulp tissue under aseptic retrieval protocols.

Storage or preservation conditions were reported in all included studies, although the procedures varied substantially. The reported approaches included immediate processing, freezing at -20°C or -80°C, and preservation or transportation in Tris-EDTA buffer, reduced transport fluid, phosphate-buffered saline, specialized transport media, or DNA/RNA Shield-type solutions.

The full-text reassessment identified explicit contamination-monitoring controls in 24 of the 30 studies (80.0%). These included operative-field or external-surface sterility controls, procedural or sampling controls, extraction blanks, no-template amplification controls, reagent or library controls, and mock positive controls. Six studies described preventive aseptic measures but did not report a specific contamination-control sample or blank within the sampling, DNA extraction, amplification, library preparation, or sequencing workflow. The type and extent of contamination monitoring varied considerably, with the most comprehensive multistage control schemes generally reported in recent sampling-comparison, protocol-validation, or low-biomass microbiome studies (Table 2, Figure 4).

*DNA extraction workflows and quality control*

All included studies reported at least some information on DNA extraction; however, the completeness of reporting varied substantially, and several studies did not specify the exact kit, manufacturer, or protocol conditions. Qiagen-based workflows predominated, particularly those using the QIAamp DNA Mini Kit, DNeasy products, or other incompletely specified Qiagen protocols. Less frequently reported approaches included PowerSoil, Norgen bacterial DNA extraction kits, magnetic bead-based methods, phenol-assisted protocols, and other customized extraction procedures. REPLI-g multiple displacement amplification was additionally used in some studies after DNA extraction.

The extraction workflow was influenced by the sample matrix. Paper-point- or file-derived root canal samples were generally processed using commercial extraction kits, sometimes combined with bead beating, enzymatic digestion, or thermal incubation. Hard-tissue and apical specimens more frequently required cryogrinding, cryopulverization, or other forms of mechanical disruption before DNA purification. Pulp-tissue studies used commercial Qiagen-based methods or customized thermomechanical protocols.

DNA quality-control reporting was heterogeneous. Reported measures included DNA concentration, purity ratios, fluorometric quantification, gel electrophoresis, Bioanalyzer assessment, qPCR screening, and sequencing-depth metrics. However, few studies reported a comprehensive and standardized set of quality metrics covering the complete pre-sequencing workflow. qPCR was used in a minority of studies to estimate bacterial load or determine sample suitability before sequencing. Host DNA depletion or microbial enrichment was rarely reported, despite its potential relevance for pulp tissue and other host-rich matrices (Table 3).

*Sequencing and bioinformatic processing*

Sequencing targets, platforms, and bioinformatic workflows varied substantially across the 28 studies that generated NGS datasets. The V3-V4 region was the most frequently targeted region of the 16S rRNA gene, followed by V4, V3-V5, V1-V3, V1-V2, and other amplicon targets. One study used full-length 16S rRNA gene sequencing, whereas another used shotgun metagenomic sequencing.

Earlier studies predominantly used operational taxonomic unit (OTU)-based approaches, commonly implemented through Mothur or QIIME. More recent studies increasingly applied amplicon sequence variant (ASV)-based workflows using DADA2, either independently or within QIIME2. The reference databases used for taxonomic assignment included HOMD/eHOMD, SILVA, Greengenes, and RDP, either individually or in combination. However, some studies did not clearly report the bioinformatic pipeline or reference database used.

This analytical heterogeneity limits direct comparisons across studies because taxonomic identification, resolution, contaminant detection, and relative-abundance estimates may be influenced by the selected target region, sequencing platform, bioinformatic pipeline, filtering criteria, and reference database (Table 4).

## Discussion

*Principal findings*

This scoping review indicates that the expansion of NGS in endodontic microbiology has outpaced standardization of the pre-analytical workflow. Although recent studies have broadened the understanding of infected pulp, primary apical periodontitis and post-treatment disease, methodological decisions made before sequencing - including sampling device, sample matrix, lysis strategy, DNA extraction kit, contamination controls and quality assessment - varied substantially across the mapped evidence.

A major message emerging from the evidence is that specimen type cannot be treated as interchangeable. Paper points and endodontic files primarily recover intracanal contents, whereas cryopulverized apices, dentin powders, periapical lesions and inflamed pulp tissues represent distinct ecological and technical matrices. These matrices differ in biomass, host-cell content, mineralized tissue burden, biofilm architecture and likelihood of environmental or reagent contamination. Therefore, differences in reported microbial composition may partly reflect biological variation and partly methodological variation, a concern also emphasized in broader discussions of root canal microbiome research [35-40].

*Contamination control and low-biomass considerations*

Contamination control is a critical consideration in endodontic NGS research because many root canal, pulpal, apical, and periapical specimens have low microbial biomass or contain a high proportion of host material. These characteristics increase their susceptibility to contamination originating from the clinical environment, sampling instruments, laboratory reagents, DNA extraction procedures, library preparation, and sample handling. Consequently, low-abundance taxa may represent genuine members of the endodontic microbiome or contaminants introduced during the pre-analytical and analytical workflow.

The present review identified explicit contamination-monitoring controls in 24 of the 30 included studies (80.0%). However, the type and extent of control varied substantially. Several studies evaluated only operative-field or external-surface sterility, whereas others included no-template amplification controls, procedural controls, extraction blanks, reagent or library controls, mock controls, or bioinformatic contaminant filtering. Only a limited number implemented controls across multiple stages of the workflow. The remaining six studies described preventive aseptic procedures but did not report a specific contamination-control sample or blank. Therefore, the presence of a control should not necessarily be interpreted as evidence of comprehensive contamination monitoring.

Future endodontic NGS studies should clearly report the stage at which each control was introduced, whether controls underwent the same extraction, amplification, library preparation, and sequencing procedures as the clinical samples, and how potential contaminants were identified and removed during bioinformatic processing. The results obtained from negative controls should also be incorporated into the interpretation of low-abundance taxa, particularly those commonly associated with environmental, skin, reagent, or laboratory sources. These recommendations are consistent with current guidance for low-biomass microbiome research, which emphasizes planned stage-specific controls, transparent reporting, and explicit contaminant-identification strategies [41-43].

*DNA extraction and matrix-specific validation*

DNA extraction remains one of the main sources of methodological inconsistency. No single extraction workflow appears optimal for all specimen types. A protocol that performs adequately for paper-point canal samples may not yield comparable results in cryoground hard tissue or inflamed pulp tissue, where mineralized matrix components, collagen, host cells and inflammatory mediators may interfere with lysis and purification. Thermomechanical, enzymatic and bead-beating steps may improve yield, but they may also introduce different biases in recovery of Gram-positive, Gram-negative, archaeal or fungal components. Host DNA depletion may become increasingly relevant as shotgun metagenomics expands in endodontics, although depletion protocols can introduce their own biases and require validation [44,45].

From a biological standpoint, the mapped studies support an ecological rather than a single-pathogen model of endodontic disease. Recurrent taxa such as Fusobacterium, Prevotella, Porphyromonas, Streptococcus, Parvimonas, Olsenella and Synergistetes-related organisms are repeatedly observed, but community profiles vary across patients, infection types, anatomical sites and sequencing workflows. This heterogeneity reinforces the need to interpret microbial signatures as community-level patterns rather than isolated species lists.

*Implications for future research*

The next advance in this field will depend less on generating more sequencing data and more on generating comparable and biologically interpretable data. Future studies should include standardized metadata, rigorous contamination monitoring, matrix-specific extraction validation, transparent reporting of DNA quality and yield, and harmonized sequencing and bioinformatic workflows whenever possible. Minimum reporting sets for endodontic microbiome studies should include the clinical diagnosis, sample source, sampling device, field disinfection method, storage medium and temperature, lysis method, extraction kit or custom protocol, negative and positive controls, DNA quantification method, sequencing target, platform, pipeline, reference database and contaminant-filtering approach.

Clinically, improved methodological standardization may support a transition from descriptive microbiome surveys toward more reproducible diagnostic and therapeutic applications. Recent studies exploring clinical parameters, periapical lesions, secondary infections, microbial interrelationships and dentin-pulp regeneration contexts show that the field is moving toward greater clinical integration [46-50]. However, without pre-analytical comparability, apparent associations between taxa and clinical status may remain difficult to reproduce.

*Strengths and limitations*

This review focused specifically on the pre-analytical and DNA extraction phases, an area that is often underemphasized in endodontic microbiome syntheses. It included clinical, surgical, *ex vivo* and methodological studies, allowing a broad map of the workflows used across infected pulp, intraradicular, apical and periapical matrices. The main limitation is that several primary studies did not report sufficient technical detail to permit fine-grained comparison of specific extraction parameters. In addition, no formal risk-of-bias appraisal was performed, which is consistent with scoping review methodology but limits conclusions about study quality or effect.

## Conclusions

This scoping review shows that, in human endodontic infection contexts, NGS-based microbial profiling has been performed using heterogeneous pre-analytical and DNA extraction workflows. Most studies relied on short-read 16S amplicon sequencing and Illumina-based platforms, but substantial variability was observed in specimen type, sampling procedures, storage conditions, lysis strategies, extraction kits, contamination controls, quality assessment, sequencing targets and bioinformatic processing. The mapped evidence indicates that operational steps and performance metrics were inconsistently reported, particularly regarding contamination monitoring, DNA quality assessment, qPCR pre-screening, host DNA depletion and matrix-specific extraction validation. Therefore, no single pre-analytical or extraction workflow can currently be considered standardized for all endodontic matrices. Future endodontic microbiome studies should prioritize standardized and matrix-specific reporting, validated extraction protocols for hard-tissue and host-rich samples, systematic negative controls and transparent quality metrics. These methodological improvements are essential to generate reproducible, comparable and clinically interpretable sequencing-based evidence in endodontics. 

## Supplementary Material

Supplementary 1

Supplementary 2

## Figures and Tables

**Table 1:** Table 1: General characteristics of the included studies (n=30).

**Study**	**Year**	**Design**	**Clinical context**	**Sample source**	**Sequencing strategy**	**Platform**
Donnermeyer *et al*., 2025 [7]	2025	Methodological study	Primary apical periodontitis	Root canal samples (paper points and files)	16S amplicon	Illumina MiSeq
Shetty *et al*., 2025 [8]	2025	Protocol validation	Irreversible pulpitis / infected dental pulp	Pulp tissue	Extraction validation	No sequencing
Hsiao *et al*., 2012 [13]	2012	Cross-sectional	Acute endodontic infections	Oral cavity, root canal and abscess samples	16S amplicon	454 pyrosequencing
Sánchez-Sanhueza *et al*., 2018 [14]	2018	Cross-sectional	Persistent endodontic infections	Root canal samples	16S amplicon	Illumina MiSeq
Keskin *et al*., 2017 [15]	2017	Comparative *ex vivo*	Primary vs secondary/persistent infections	Cryogenically ground whole roots	16S amplicon	454 pyrosequencing
Shaji *et al*., 2026 [16]	2026	Cross-sectional	Symptomatic apical periodontitis	Pulpal samples	Full-length 16S	Oxford Nanopore
Kesim *et al*., 2023 [2]	2023	Comparative clinical	Primary vs persistent/secondary infections	Root canal samples	16S amplicon	Illumina MiSeq
Sun *et al*., 2022 [17]	2022	Paired surgical study	Persistent apical periodontitis	Root canal, extraradicular biofilm and periapical lesion samples	16S amplicon	Illumina HiSeq 2500
Zhang *et al*., 2022 [18]	2022	Paired/co-analysis	Post-treatment apical periodontitis	Root apex and periapical granulation tissue	16S amplicon	Illumina NovaSeq 6000
Li *et al*., 2025 [19]	2025	Comparative clinical	Apical periodontitis with/without T2DM	Infected root canal samples	16S amplicon	Illumina NovaSeq
Sterzenbach *et al*., 2020 [20]	2020	Method optimization	Human tooth hard tissue model	Ground dental hard tissue	DNA isolation optimization	No sequencing
Bouillaguet *et al*., 2018 [21]	2018	Comparative clinical	Primary vs secondary apical periodontitis	Intraradicular content with dentin control	16S amplicon	Illumina MiSeq
Pérez-Carrasco *et al*., 2023 [3]	2023	Paired surgical study	Persistent apical periodontitis	Root apices and periapical lesions	16S amplicon	Illumina MiSeq
Alquria *et al*., 2024a [6]	2024	Longitudinal clinical	Primary infection with apical periodontitis	Root canal samples before/after chemomechanical preparation	16S amplicon	Illumina MiSeq
Arias-Moliz *et al*., 2024 [4]	2024	Comparative surgical	Post-treatment endodontic infections	Root apices and periapical lesions	16S amplicon	Illumina MiSeq
Tawfik *et al*., 2018 [22]	2018	Cross-sectional	Primary endodontic infections	Paper points and K-file samples	16S amplicon	Illumina MiSeq
Siqueira *et al*., 2011 [23]	2011	Surgical apical sampling	Apical periodontitis	Cryopulverized apical root segments	16S amplicon	454 pyrosequencing
Saber *et al*., 2012 [24]	2012	Surgical lesion study	Periradicular lesions of endodontic origin	Periradicular lesion samples	16S amplicon	454 pyrosequencing
Kesim *et al*., 2024 [25]	2024	Longitudinal experimental	Primary apical periodontitis (two-visit treatment)	Root canal samples across S1-S4	16S amplicon	Illumina MiSeq
Zandi *et al*., 2018 [26]	2018	Retreatment longitudinal	Root-filled teeth with apical periodontitis	Root canal samples before/after irrigation	16S amplicon	454 pyrosequencing
Zahran *et al*., 2021 [27]	2021	Cross-sectional	Irreversible pulpitis	Inflamed pulp tissue sampled intracanal	16S amplicon	Illumina MiSeq
Kruly *et al*., 2022 [28]	2022	Before-after clinical	Primary and secondary infections	Root canal samples before and after chemomechanical preparation	16S amplicon	Illumina MiSeq
Zahran *et al*., 2022 [29]	2022	Randomized protocol study	Molar root canal treatment	Molar root canals before/after preparation	16S amplicon	Illumina MiSeq
Alquria *et al*., 2024b [5]	2024	Cross-sectional clinical	Primary infection with apical periodontitis	Root canal samples	16S amplicon	Illumina MiSeq
Siqueira *et al*., 2016 [30]	2016	Surgical apical sampling	Post-treatment apical periodontitis	Cryopulverized apical root samples	16S amplicon	Illumina MiSeq
Ping *et al*., 2015 [31]	2015	Surgical apical sampling	Persistent apical periodontitis	Cryogenically ground apical root sections	16S amplicon	454 pyrosequencing
Amaral *et al*., 2024 [32]	2024	Interventional longitudinal	Primary asymptomatic apical periodontitis	Root canal samples S1-S3	16S amplicon	Illumina MiSeq
Qian *et al*., 2019 [33]	2019	Comparative with controls	Apical periodontitis with/without RCT and healthy controls	Apical root samples	16S amplicon	Illumina MiSeq
Shetty *et al*., 2026 [9]	2026	Shotgun metagenomic study	Chronic irreversible pulpitis	Pulp tissue	Shotgun metagenomics	Illumina HiSeq
Park *et al*., 2025 [34]	2025	Paired-site study	Secondary/persistent infections	Root canal and gingival sulcus samples	16S amplicon	Illumina MiSeq

NGS: Next-generation sequencing; T2DM: Type 2 diabetes mellitus; RCT: Root canal treatment.

**Table 2:** Table 2: Sampling, field preparation, storage and contamination-control features.

**Study**	**Asepsis/disinfection**	**Sample procurement**	**Storage/preservation**	**Contamination control**
Donnermeyer *et al*., 2025 [7]	Rubber dam + surface disinfection	Root canal samples (paper points and files)	Reported, details heterogeneous	Unused sterile paper points and files; extraction/library monitoring; mock positive controls.
Shetty *et al*., 2025 [8]	Rubber dam + aseptic pulp retrieval	Pulp tissue	Frozen at -20°C	Negative controls processed with every DNA-extraction batch.
Hsiao *et al*., 2012 [13]	Rubber dam + surface disinfection	Oral cavity, root canal and abscess samples	Frozen at -20°C	No-template negative controls included for every barcoded primer pair.
Sánchez-Sanhueza *et al*., 2018 [14]	Rubber dam + surface disinfection	Root canal samples	RTF medium	Operative-field sterility samples; cases accepted only when culture remained negative.
Keskin *et al*., 2017 [15]	Aseptic surgical/clinical field	Cryogenically ground whole roots	TE buffer	Disinfected healthy-tooth control sample; no quantifiable DNA and negative sequencing result.
Shaji *et al*., 2026 [16]	Rubber dam + surface disinfection	Pulpal samples	Frozen at -80°C	Not explicitly reported.
Kesim *et al*., 2023 [2]	Rubber dam + surface disinfection	Root canal samples	Reported, details heterogeneous	Access-cavity control samples used to verify field disinfection; controls were PCR-negative.
Sun *et al*., 2022 [17]	Reported, details variable	Root canal, extraradicular biofilm and periapical lesion samples	Frozen at -20°C	Periosteal tissue collected as a sampling-contamination control; bacterial DNA was not detected.
Zhang *et al*., 2022 [18]	Reported, details variable	Root apex and periapical granulation tissue	Frozen at -20°C	Not explicitly reported.
Li *et al*., 2025 [19]	Rubber dam + surface disinfection	Infected root canal samples	Frozen at -80°C	Not explicitly reported.
Sterzenbach *et al*., 2020 [20]	Laboratory model	Ground dental hard tissue	Frozen at -80°C	Not explicitly reported.
Bouillaguet *et al*., 2018 [21]	Aseptic surgical/clinical field	Intraradicular content with dentin control	Immediate processing	Negative controls were processed and sequenced alongside dentin and root-canal samples.
Pérez-Carrasco *et al*., 2023 [3]	Aseptic surgical/clinical field	Root apices and periapical lesions	Frozen at -20°C	Outer-root-surface control sample collected after disinfection.
Alquria *et al*., 2024a [6]	Rubber dam + surface disinfection	Root canal samples before/after chemomechanical preparation	DNA/RNA Shield	Water negative controls underwent the same DNA-isolation and amplification procedures.
Arias-Moliz *et al*., 2024 [4]	Aseptic surgical/clinical field	Root apices and periapical lesions	Frozen at -20°C	Extraction-batch, disinfection, and negative PCR controls were amplified and sequenced.
Tawfik *et al*., 2018 [22]	Reported, details variable	Paper points and K-file samples	Immediate processing	Unused sterile file/paper points and elution solution used as negative controls.
Siqueira *et al*., 2011 [23]	Surgical/extraction sampling + external decontamination	Cryopulverized apical root segments	Frozen at -80°C	External-root-surface sterility controls; all were PCR-negative.
Saber *et al*., 2012 [24]	Reported, details variable	Periradicular lesion samples	Immediate processing	Sterile-water no-template negative control included in every PCR run.
Kesim *et al*., 2024 [25]	Rubber dam + surface disinfection	Root canal samples across S1-S4	Frozen at -80°C	Operative-field sterility controls collected and tested by PCR; all were negative.
Zandi *et al*., 2018 [26]	Rubber dam + surface disinfection	Root canal samples before/after irrigation	TE buffer	Access-cavity sterility controls; samples with positive controls were excluded.
Zahran *et al*., 2021 [27]	Rubber dam + aseptic pulp retrieval	Inflamed pulp tissue sampled intracanal	Immediate processing	Contamination-control samples, sterile paper-point controls, and no-template controls were sequenced.
Kruly *et al*., 2022 [28]	Reported, details variable	Root canal samples before and after chemomechanical preparation	TE buffer	Not explicitly reported.
Zahran *et al*., 2022 [29]	Rubber dam + surface disinfection	Molar root canals before/after preparation	Immediate processing	Tooth-surface, negative sampling, and no-extraction controls included in NGS analysis.
Alquria *et al*., 2024b [5]	Rubber dam + surface disinfection	Root canal samples	Transport/stored at 4°C	Sterility-control samples plus water negative controls; decontam used analytically.
Siqueira *et al*., 2016 [30]	Rubber dam + surface disinfection	Cryopulverized apical root samples	TE buffer	Outer-root-surface sterility controls; all were PCR-negative.
Ping *et al*., 2015 [31]	Surgical/extraction sampling + external decontamination	Cryogenically ground apical root sections	Immediate processing	Negative amplification controls; human DNA was used as a positive control.
Amaral *et al*., 2024 [32]	Rubber dam + surface disinfection	Root canal samples S1-S3	Immediate processing	Cavosurface sterility controls; inclusion required a negative control result.
Qian *et al*., 2019 [33]	Surgical/extraction sampling + external decontamination	Apical root samples	Frozen at -20°C	Not explicitly reported.
Shetty *et al*., 2026 [9]	Rubber dam + aseptic pulp retrieval	Pulp tissue	Frozen at -80°C	Procedural, extraction, and reagent/library negative controls processed and sequenced.
Park *et al*., 2025 [34]	Rubber dam + surface disinfection	Root canal and gingival sulcus samples	Frozen at -80°C	Untreated paper points and pure reagents used as negative controls; decontam filtering applied.

Contamination control was considered reported only when the full text identified a sampling/field control, procedural blank, extraction blank, no-template amplification control, reagent/library control, or another explicitly described contamination-monitoring sample. General aseptic measures alone were not classified as contamination controls.

**Table 3:** Table 3: DNA extraction workflows, matrix pretreatment and pre-sequencing quality control.

**Study**	**Extraction workflow**	**Matrix pretreatment / lysis**	**DNA QC metrics**	**qPCR pre-screening**
Donnermeyer *et al*., 2025 [7]	QIAamp DNA Mini Kit (Qiagen)	Cryogrinding/pulverization	Qubit, DNA yield/concentration	No
Shetty *et al*., 2025 [8]	Qiagen kit/protocol (unspecified)	Thermal + mechanical disruption	Qubit, NanoDrop, Purity ratio, DNA yield/concentration	No
Hsiao *et al*., 2012 [13]	Custom or not clearly specified	Bead-beating/homogenization	Gel electrophoresis, DNA yield/concentration	No
Sánchez-Sanhueza *et al*., 2018 [14]	QIAamp DNA Mini Kit (Qiagen)	Cryogrinding/pulverization	NanoDrop, Gel electrophoresis	No
Keskin *et al*., 2017 [15]	Norgen bacterial DNA extraction kit	Cryogrinding/pulverization; Bead-beating/homogenization; Enzymatic digestion	Qubit, NanoDrop, Gel electrophoresis, DNA yield/concentration	No
Shaji *et al*., 2026 [16]	Custom or not clearly specified	Not explicitly detailed	DNA yield/concentration	No
Kesim *et al*., 2023 [2]	Custom or not clearly specified	Cryogrinding/pulverization; Thermal incubation	DNA yield/concentration	No
Sun *et al*., 2022 [17]	QIAamp DNA Mini Kit (Qiagen)	Cryogrinding/pulverization	Qubit, Gel electrophoresis, Bioanalyzer	No
Zhang *et al*., 2022 [18]	DNeasy kit	Enzymatic digestion; Thermal incubation	NanoDrop, DNA yield/concentration	No
Li *et al*., 2025 [19]	Qiagen kit/protocol (unspecified)	Not explicitly detailed	NanoDrop, Gel electrophoresis, Bioanalyzer, DNA yield/concentration	No
Sterzenbach *et al*., 2020 [20]	Custom or not clearly specified	Cryogrinding/pulverization; Bead-beating/homogenization; Enzymatic digestion; Thermal incubation	DNA yield/concentration	Yes
Bouillaguet *et al*., 2018 [21]	Custom or not clearly specified	Cryogrinding/pulverization	Bioanalyzer, DNA yield/concentration	Yes
Pérez-Carrasco *et al*., 2023 [3]	Qiagen kit/protocol (unspecified)	Cryogrinding/pulverization; Enzymatic digestion	NanoDrop	No
Alquria *et al*., 2024a [6]	Qiagen kit/protocol (unspecified)	Cryogrinding/pulverization; Bead-beating/homogenization	DNA yield/concentration	Yes
Arias-Moliz *et al*., 2024 [4]	Qiagen kit/protocol (unspecified)	Cryogrinding/pulverization; Enzymatic digestion	NanoDrop, DNA yield/concentration	No
Tawfik *et al*., 2018 [22]	PowerSoil DNA isolation kit	Cryogrinding/pulverization; Bead-beating/homogenization	Qubit, NanoDrop, Gel electrophoresis, Bioanalyzer, DNA yield/concentration	No
Siqueira *et al*., 2011 [23]	QIAamp DNA Mini Kit (Qiagen)	Cryogrinding/pulverization; Enzymatic digestion	NanoDrop, Gel electrophoresis, DNA yield/concentration	No
Saber *et al*., 2012 [24]	Custom or not clearly specified	Not explicitly detailed	Gel electrophoresis, DNA yield/concentration	No
Kesim *et al*., 2024 [25]	Custom or not clearly specified	Cryogrinding/pulverization; Bead-beating/homogenization	Gel electrophoresis, DNA yield/concentration	No
Zandi *et al*., 2018 [26]	Custom or not clearly specified	Cryogrinding/pulverization; Bead-beating/homogenization	Bioanalyzer, DNA yield/concentration	No
Zahran *et al*., 2021 [27]	Qiagen extraction + REPLI-g MDA	Bead-beating/homogenization	Qubit, Gel electrophoresis, DNA yield/concentration	Yes
Kruly *et al*., 2022 [28]	Qiagen kit/protocol (unspecified)	Cryogrinding/pulverization; Bead-beating/homogenization	Qubit, Gel electrophoresis, DNA yield/concentration	No
Zahran *et al*., 2022 [29]	Qiagen extraction + REPLI-g MDA	Cryogrinding/pulverization; Thermal incubation	Gel electrophoresis, DNA yield/concentration	Yes
Alquria *et al*., 2024b [5]	Qiagen kit/protocol (unspecified)	Cryogrinding/pulverization; Bead-beating/homogenization	DNA yield/concentration	Yes
Siqueira *et al*., 2016 [30]	QIAamp DNA Mini Kit (Qiagen)	Cryogrinding/pulverization; Bead-beating/homogenization	DNA yield/concentration	No
Ping *et al*., 2015 [31]	Magnetic bead-based extraction	Cryogrinding/pulverization; Bead-beating/homogenization	NanoDrop, Gel electrophoresis, Bioanalyzer, Purity ratio, DNA yield/concentration	No
Amaral *et al*., 2024 [32]	Custom thermomechanical/phenol-assisted protocol	Thermal + mechanical disruption	Read-depth/sequence QC only	No
Qian *et al*., 2019 [33]	Custom or not clearly specified	Cryogrinding/pulverization; Bead-beating/homogenization	NanoDrop, Gel electrophoresis, DNA yield/concentration	No
Shetty *et al*., 2026 [9]	QIAamp DNA Mini Kit (Qiagen)	Cryogrinding/pulverization; Bead-beating/homogenization; Enzymatic digestion	Qubit, Gel electrophoresis, DNA yield/concentration	No
Park *et al*., 2025 [34]	PowerSoil DNA isolation kit	Cryogrinding/pulverization; Bead-beating/homogenization	Gel electrophoresis, Purity ratio, DNA yield/concentration	No

QC: Quality control; qPCR: Quantitative polymerase chain reaction; MDA: Multiple displacement amplification.

**Table 4:** Table 4: Sequencing targets, platforms and bioinformatic processing workflows.

**Study**	**Target region / type**	**Platform**	**Processing pipeline**	**Reference database**
Donnermeyer *et al*., 2025 [7]	V4	Illumina MiSeq	DADA2/ASV, Cutadapt	HOMD/eHOMD, SILVA
Shetty *et al*., 2025 [8]	Not applicable	No sequencing	Not applicable	Not applicable
Hsiao *et al*., 2012 [13]	V1-V2	454 pyrosequencing	Mothur	HOMD/eHOMD, SILVA, RDP
Sánchez-Sanhueza *et al*., 2018 [14]	V3-V4	Illumina MiSeq	QIIME/QIIME2, Mothur	Greengenes
Keskin *et al*., 2017 [15]	V4	454 pyrosequencing	Not clear	Not clear
Shaji *et al*., 2026 [16]	Full-length 16S	Oxford Nanopore	QIIME/QIIME2	Not clear
Kesim *et al*., 2023 [2]	V3-V4	Illumina MiSeq	DADA2/ASV, QIIME/QIIME2	Greengenes
Sun *et al*., 2022 [17]	V3-V4	Illumina HiSeq 2500	QIIME/QIIME2	HOMD/eHOMD, SILVA
Zhang *et al*., 2022 [18]	V3-V4	Illumina NovaSeq 6000	QIIME/QIIME2	SILVA
Li *et al*., 2025 [19]	V3-V4	Illumina NovaSeq	Mothur	SILVA
Sterzenbach *et al*., 2020 [20]	Not applicable	No sequencing	Not applicable	Not applicable
Bouillaguet *et al*., 2018 [21]	V3-V4	Illumina MiSeq	Mothur	HOMD/eHOMD, Greengenes
Pérez-Carrasco *et al*., 2023 [3]	V3-V4	Illumina MiSeq	Mothur	SILVA
Alquria *et al*., 2024a [6]	V3-V4	Illumina MiSeq	DADA2/ASV	HOMD/eHOMD, SILVA, RDP
Arias-Moliz *et al*., 2024 [4]	V3-V4	Illumina MiSeq	Mothur	SILVA
Tawfik *et al*., 2018 [22]	V3-V4	Illumina MiSeq	Mothur	SILVA, Greengenes
Siqueira *et al*., 2011 [23]	968f-1401r amplicon	454 pyrosequencing	Not clear	SILVA
Saber *et al*., 2012 [24]	V1-V2	454 pyrosequencing	QIIME/QIIME2	Not clear
Kesim *et al*., 2024 [25]	V3-V4	Illumina MiSeq	DADA2/ASV, QIIME/QIIME2	SILVA, Greengenes
Zandi *et al*., 2018 [26]	V3-V5	454 pyrosequencing	QIIME/QIIME2	HOMD/eHOMD, SILVA
Zahran *et al*., 2021 [27]	V3-V5	Illumina MiSeq	QIIME/QIIME2	HOMD/eHOMD, Greengenes
Kruly *et al*., 2022 [28]	V3-V4	Illumina MiSeq	DADA2/ASV, QIIME/QIIME2	SILVA, Greengenes
Zahran *et al*., 2022 [29]	V3-V4	Illumina MiSeq	QIIME/QIIME2	HOMD/eHOMD
Alquria *et al*., 2024b [5]	V3-V4	Illumina MiSeq	DADA2/ASV	HOMD/eHOMD, SILVA, RDP
Siqueira *et al*., 2016 [30]	V4	Illumina MiSeq	Mothur	HOMD/eHOMD, SILVA
Ping *et al*., 2015 [31]	V1-V3	454 pyrosequencing	Mothur	HOMD/eHOMD, SILVA, RDP
Amaral *et al*., 2024 [32]	V3-V4	Illumina MiSeq	Mothur	SILVA
Qian *et al*., 2019 [33]	V3-V4	Illumina MiSeq	QIIME/QIIME2	Greengenes
Shetty *et al*., 2026 [9]	Shotgun metagenome	Illumina HiSeq	Cutadapt, WGSA2	SILVA
Park *et al*., 2025 [34]	V3-V4	Illumina MiSeq	DADA2/ASV, QIIME/QIIME2, Cutadapt	SILVA

**Figure 1:**
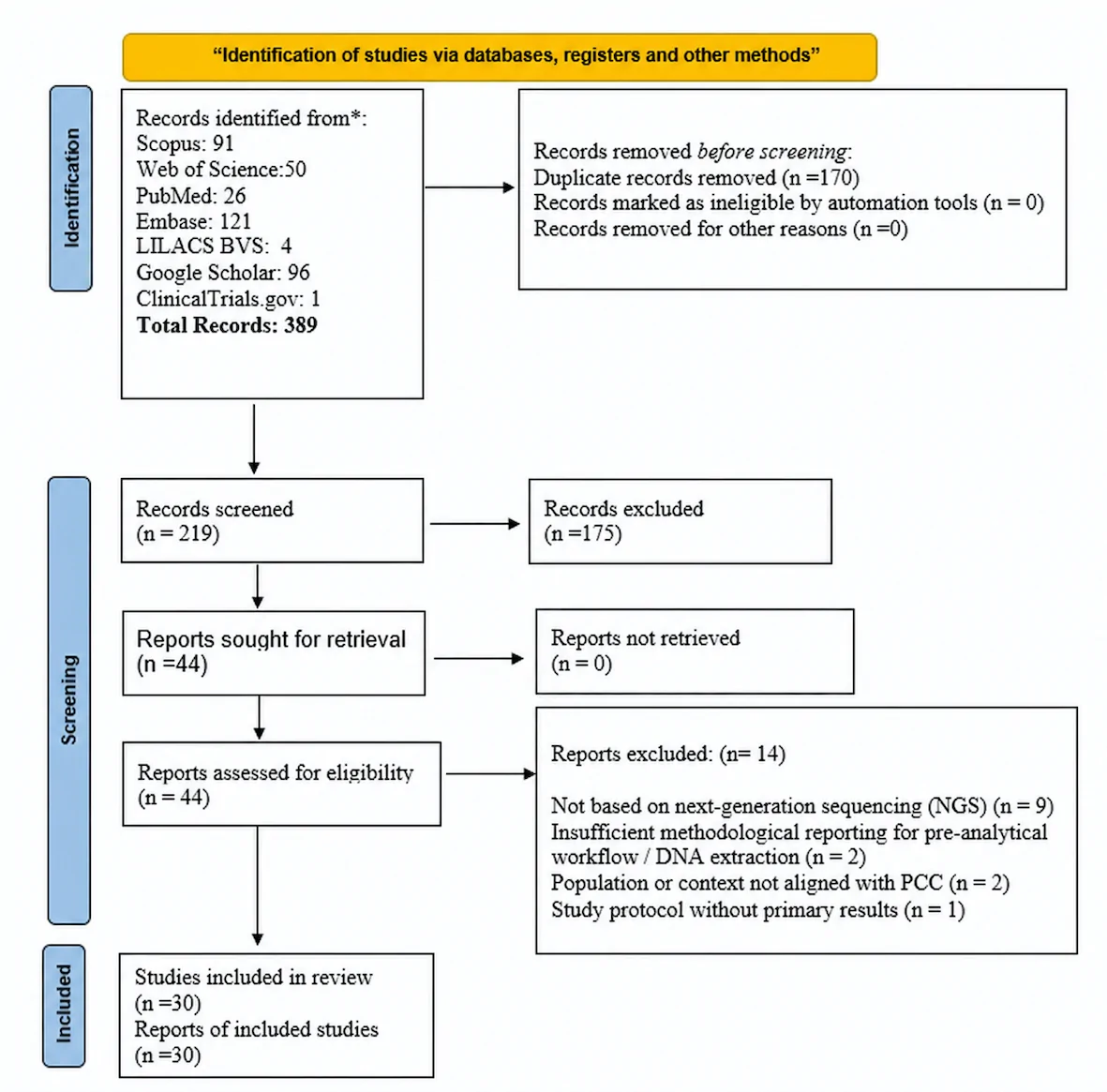
Fig. 1: Flow diagram of the study-selection process according to PRISMA-ScR.

**Figure 2:**
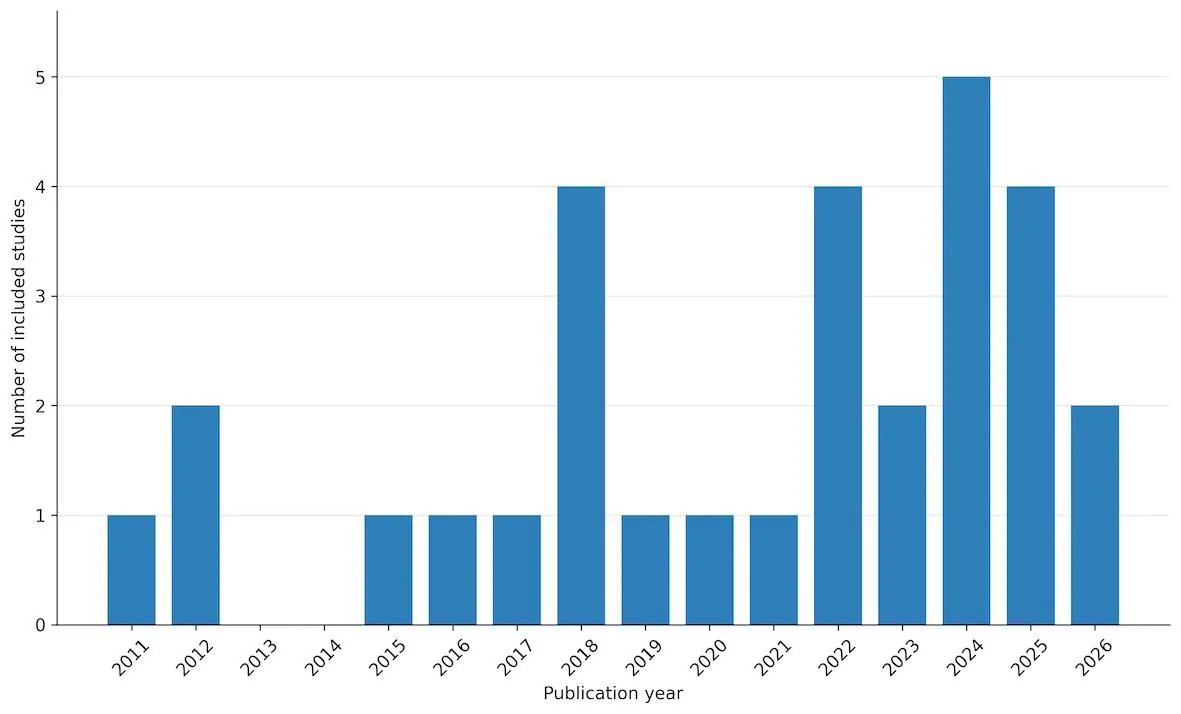
Fig. 2: Publication trend of the 30 included studies from 2011 to March 23, 2026. Data for 2026 represent a partial year.

**Figure 3:**
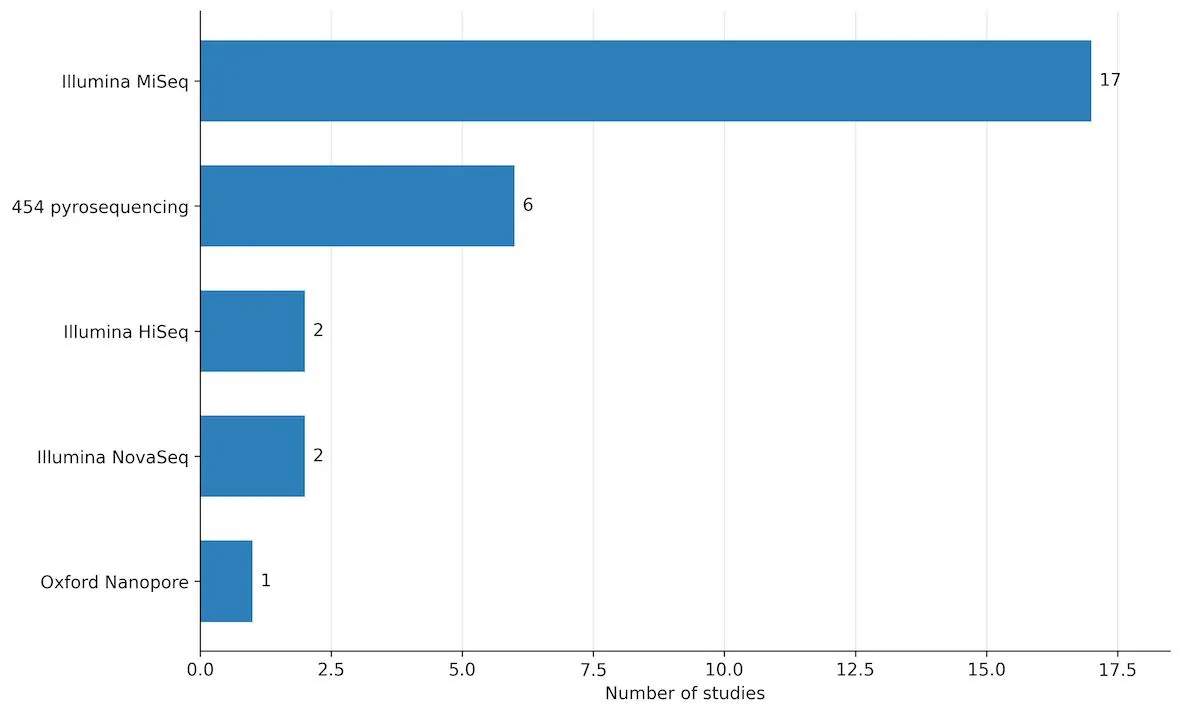
Fig. 3: Sequencing platforms used in the 28 included studies that generated NGS datasets. Two methodological DNA-extraction studies did not generate sequencing datasets and were therefore not represented in the figure.

**Figure 4:**
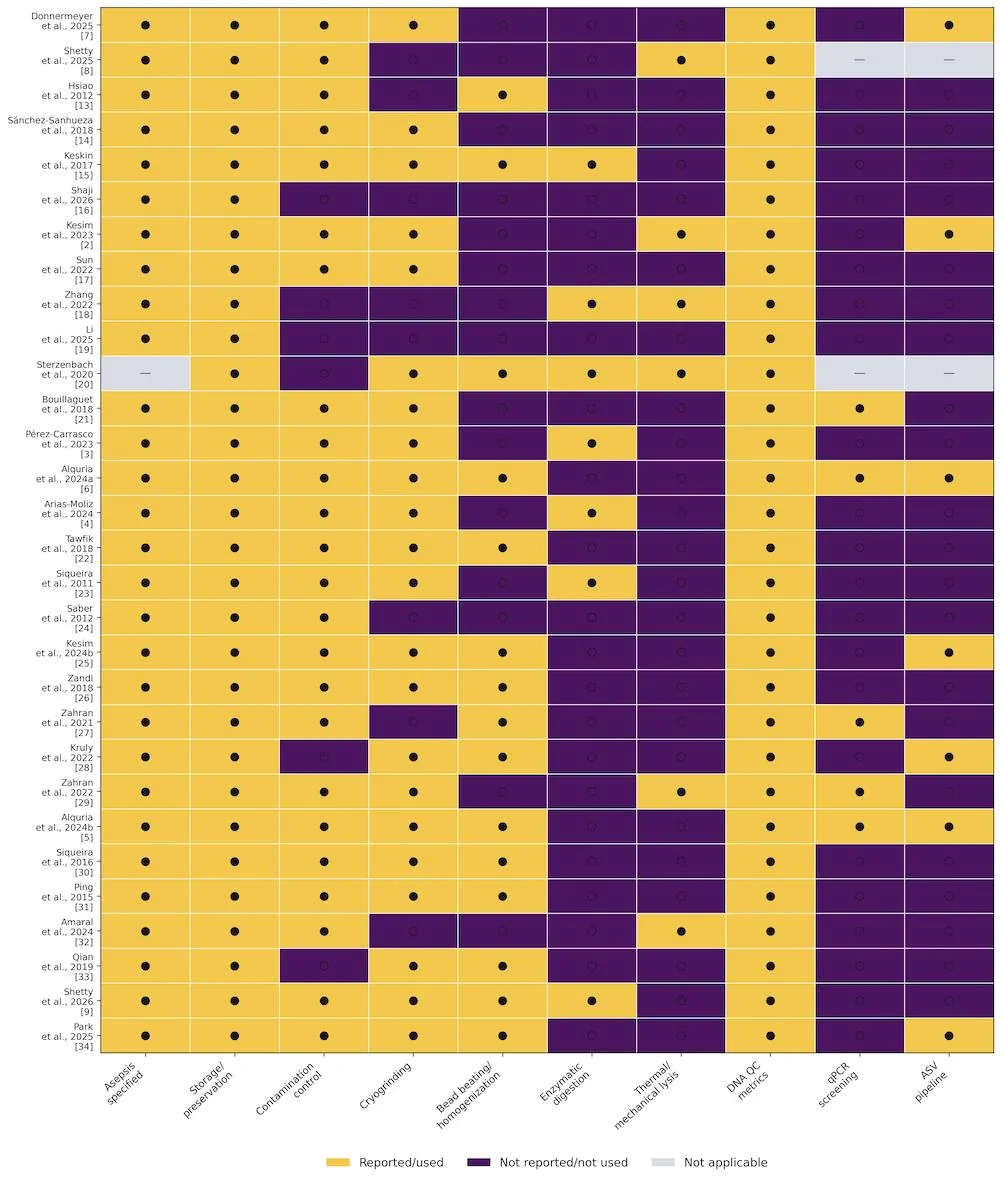
Fig. 4: Evidence map of workflow reporting across the included studies. Filled circles indicate reported features, open circles indicate unreported features, and dashes indicate not applicable.

## Data Availability

Declared none.
