# Supplementary material for: DNA extraction methods and pre-analytical workflows for next-generation sequencing in infected dental pulp and endodontic infections: A scoping review
Source: J Clin Exp Dent. 2026 Sep 15;18(10):e1181–97. doi: 10.4317/jced.64251 (PMC13739084; doi:10.4317/jced.64251)
Supplement: Supplementary file 1 [file jced-18-10-e1181-s001.pdf]

Search strategies used for the scoping review

Supplementary Table S1. Electronic database search strategies and records retrieved.

Database	Records identified	Full search strategy
Scopus	91	(TITLE-ABS-KEY (("next generation sequencing" OR NGS OR "high-throughput sequencing" OR "massively parallel sequencing" OR metagenom* OR metatranscriptom* OR "16S rRNA" OR amplicon* OR "shotgun sequencing" OR "whole genome sequencing" OR WGS OR nanopore OR Illumina OR MiSeq OR NovaSeq OR "Ion Torrent" OR MinION OR pyrosequenc* OR 454 OR "Roche 454") AND (("DNA" W/2 (extract* OR isolat* OR purif*)) OR "nucleic acid extraction" OR "sample preparation" OR "sample prep" OR pre-analytical OR preanalytical OR lysis OR "cell lysis" OR "bead beat*" OR "bead-beat*" OR "mechanical lysis" OR "thermal lysis" OR thermomechanical OR enzymatic OR "host DNA depletion" OR "human DNA depletion" OR "inhibitor removal" OR inhibitor*) AND ((("dental pulp" OR pulp) W/3 (infect* OR necros* OR pulpitis OR inflam*)) OR ("root canal" OR endodont* OR "endodontic infection*" OR "apical periodontitis" OR "periapical lesion*" OR "periradicular lesion*" OR "endodontic microbiome" OR "endodontic microbiota"))) AND NOT TITLE-ABS-KEY (saliva OR plaque OR subgingival OR periodontal OR gingivitis OR caries)) AND PUBYEAR > 2010
Web of Science Core Collection	50	TS=(("next generation sequencing" OR NGS OR "high-throughput sequencing" OR "massively parallel sequencing" OR metagenom* OR metatranscriptom* OR "16S rRNA" OR amplicon* OR "shotgun sequencing" OR "whole genome sequencing" OR WGS OR nanopore OR Illumina OR MiSeq OR NovaSeq OR "Ion Torrent" OR MinION OR pyrosequenc* OR 454 OR "Roche 454") AND ((DNA NEAR/2 (extract* OR isolat* OR purif*)) OR "nucleic acid extraction" OR "sample preparation" OR "sample prep" OR pre-analytical OR preanalytical OR lysis OR "cell lysis" OR "bead beat*" OR "bead-beat*" OR "mechanical lysis" OR "thermal lysis" OR thermomechanical OR enzymatic OR "host DNA depletion" OR "human DNA depletion" OR "inhibitor removal" OR inhibitor*) AND ((("dental pulp" OR pulp) NEAR/3 (infect* OR necros* OR pulpitis OR inflam*)) OR ("root canal" OR endodont* OR "endodontic infection*" OR "apical periodontitis" OR "periapical lesion*" OR "periradicular lesion*" OR "endodontic microbiome" OR "endodontic microbiota"))) NOT TS=(saliva OR plaque OR subgingival OR periodontal OR gingivitis OR caries) AND PY=(2011-2026)
Embase	121	(("next generation sequencing" OR NGS OR metagenom* OR metatranscriptom* OR "16S rRNA" OR amplicon* OR "shotgun sequencing" OR "whole genome sequencing" OR WGS OR nanopore OR Illumina OR pyrosequenc* OR 454 OR "Roche 454") AND ("dna extract*" OR "dna isolat*" OR "dna purif*" OR "nucleic acid extract*" OR "sample preparation" OR "sample prep" OR preanalyt* OR lysis OR "cell lysis" OR "bead beat*" OR "bead-beat*" OR "mechanical lysis" OR "thermal lysis" OR thermomechanical OR enzymatic OR "host dna depletion" OR "human dna depletion" OR "inhibitor removal" OR inhibitor*) AND ("dental pulp" OR "infected pulp" OR "necrotic pulp" OR pulpitis OR endodont* OR "root canal" OR "endodontic infection*" OR "apical periodontitis" OR periapical OR periradicular OR "endodontic microbiome" OR "endodontic microbiota")) NOT (saliva OR plaque OR subgingival OR periodontal OR gingivitis OR caries)
PubMed/MEDLINE	26	(("High-Throughput Nucleotide Sequencing"[Mesh] OR "Metagenomics"[Mesh] OR "Sequencing, DNA"[Mesh] OR "next generation sequencing"[tiab] OR NGS[tiab] OR "high-throughput sequencing"[tiab] OR "massively parallel sequencing"[tiab] OR metagenom*[tiab] OR metatranscriptom*[tiab] OR "16S rRNA"[tiab] OR amplicon*[tiab] OR "shotgun sequencing"[tiab] OR "whole genome sequencing"[tiab] OR WGS[tiab] OR nanopore[tiab] OR Illumina[tiab] OR MiSeq[tiab] OR NovaSeq[tiab] OR "Ion Torrent"[tiab] OR MinION[tiab] OR pyrosequenc*[tiab] OR 454[tiab] OR "Roche 454"[tiab]) AND ("Nucleic Acid Extraction Techniques"[Mesh] OR "DNA extraction"[tiab] OR "DNA isolation"[tiab] OR "DNA purification"[tiab] OR "nucleic acid extraction"[tiab] OR "sample preparation"[tiab] OR "sample prep"[tiab] OR preanalyt*[tiab] OR "pre-analyt*"[tiab] OR lysis[tiab] OR "cell lysis"[tiab] OR "bead beat*"[tiab] OR "bead-beat*"[tiab] OR "mechanical lysis"[tiab] OR "thermal lysis"[tiab] OR

		thermomechanical[tiab] OR enzymatic[tiab] OR "host DNA depletion"[tiab] OR "human DNA depletion"[tiab] OR "inhibitor removal"[tiab] OR inhibitor*[tiab]) AND ((("Dental Pulp"[Mesh] OR "dental pulp"[tiab] OR pulp[tiab]) AND (infect*[tiab] OR necros*[tiab] OR pulpitis[tiab] OR inflamm*[tiab])) OR ("Root Canal Therapy"[Mesh] OR "Periapical Diseases"[Mesh] OR endodont*[tiab] OR "root canal"[tiab] OR "endodontic infection*"[tiab] OR "apical periodontitis"[tiab] OR "periapical lesion*"[tiab] OR "periradicular lesion*"[tiab] OR "endodontic microbiome"[tiab] OR "endodontic microbiota"[tiab]))) NOT (saliva[tiab] OR plaque[tiab] OR subgingival[tiab] OR periodontal[tiab] OR gingivitis[tiab] OR caries[tiab]) AND ("2011/01/01"[dp] : "3000"[dp])
LILACS/BVS	4	tw:(("next generation sequencing" OR NGS OR "high-throughput sequencing" OR "massively parallel sequencing" OR "secuenciación de nueva generación" OR "secuenciación de alto rendimiento" OR "sequenciamento de nova geração" OR "sequenciamento de alto rendimento" OR metagenom* OR metatranscriptom* OR metagenómic* OR metagenômic* OR "16S rRNA" OR amplicon* OR "shotgun sequencing" OR "whole genome sequencing" OR WGS OR pyrosequenc* OR 454 OR "Roche 454")) AND tw:(("dna extract*" OR "dna isolat*" OR "dna purif*" OR "nucleic acid extract*" OR "extracción de adn" OR "aislamiento de adn" OR "purificación de adn" OR "extração de dna" OR "isolamento de dna" OR "purificação de dna" OR lysis OR lisis OR lise OR "cell lysis" OR "bead beat*" OR "bead-beat*" OR "mechanical lysis" OR "thermal lysis" OR thermomechanical OR enzimatic* OR enzimátic* OR "host dna depletion" OR "human dna depletion")) AND tw:(endodont* OR "root canal" OR "canal radicular" OR "endodontic infection*" OR "infecção endodôntica" OR "infección endodóntica" OR "apical periodontitis" OR "periodontitis apical" OR "periodontite apical" OR periapical OR periapicales OR periapicais OR "dental pulp" OR "pulpa dental" OR "polpa dentária" OR "infected pulp" OR "pulpa infectada" OR "polpa infectada" OR pulpitis) AND tw:(protocol* OR metod* OR method* OR optimiz* OR compar* OR evaluat* OR kit* OR rendimiento OR pureza OR inhibidor* OR yield) NOT tw:(saliva OR placa OR plaque OR subgingival OR periodontal OR gingivitis OR caries)

Grey literature and register search strategies and records retrieved.

**Source**	**Records identified**	**Source type**	**Full search strategy**
Google Scholar	96	Grey literature	(endodontic OR "root canal" OR "dental pulp" OR "apical periodontitis") (NGS OR "next generation sequencing" OR metagenomics OR "16S rRNA" OR "shotgun sequencing") ("DNA extraction" OR "sample preparation" OR lysis OR "host DNA depletion") (thesis OR dissertation OR tesis OR "doctoral thesis" OR "master thesis") (filetype:pdf OR site:.edu OR site:.ac OR site:dspace OR site:repository) -saliva -plaque -periodontal -caries
ClinicalTrials.gov	1	Trial register	AREA[ConditionSearch]("apical periodontitis" OR pulpitis OR "endodontic infection" OR "root canal" OR endodontic) AND AREA[BasicSearch](microbiome OR metagenomic OR metagenomics OR sequencing OR "16S" OR "16S rRNA" OR NGS OR "next generation sequencing") NOT AREA[BasicSearch](saliva OR plaque OR periodontal OR gingivitis OR caries)

Summary of sources and total records before deduplication.

**Source category**	**Source**	**Records identified**
Electronic database	Scopus	91
Electronic database	Web of Science Core Collection	50
Electronic database	PubMed/MEDLINE	26
Electronic database	Embase	121
Electronic database	LILACS/BVS	4
Grey literature	Google Scholar	96
Trial register	ClinicalTrials.gov	1
**Total**	**All sources**	**389**
