# Supplementary material for: DNA extraction methods and pre-analytical workflows for next-generation sequencing in infected dental pulp and endodontic infections: A scoping review
Source: J Clin Exp Dent. 2026 Sep 15;18(10):e1181–97. doi: 10.4317/jced.64251 (PMC13739084; doi:10.4317/jced.64251)
Supplement: Supplementary file 2 [file jced-18-10-e1181-s002.pdf]

Supplementary Table S2. Full-text assessment and eligibility decisions

Full-text assessment of studies on pre-analytical workflows and DNA extraction methods for NGS in human endodontic infection samples

Full-text reports assessed: 44 Studies included: 30 Reports excluded: 14

Decision rule: Full-text reports were assessed according to the predefined PCC framework and eligibility criteria. Studies were included when they involved human endodontic infection-related samples, used NGS or explicitly evaluated a DNA extraction workflow intended for NGS applications, and provided sufficient methodological information for evidence mapping.

Included studies (n = 30)

No.	Title	DOI	Final decision	Rationale
1	A Methodological Study on Microbial In Vivo Sampling Methods of Root Canal Microbiota for Next-Generation Gene Sequencing Analysis	10.1016/j.joen.2024.11.007	Include	Human endodontic study using NGS with useful reporting of sampling, contamination controls, DNA extraction, and DNA quality.
2	Thermomechanical DNA extraction from infected dental pulp for next-generation sequencing applications	10.1016/j.jobcr.2025.09.029	Include	Explicit validation of a DNA extraction protocol designed for NGS applications in infected human dental pulp.
3	Microbial transformation from normal oral microbiota to acute endodontic infections	10.1186/1471-2164-13-345	Include	Includes human endodontic samples and next-generation sequencing with sufficient methodological detail.
4	Metagenomic study of bacterial microbiota in persistent endodontic infections using Next-generation sequencing	10.1111/iej.12953	Include	Human persistent endodontic infection study with NGS and adequate reporting of pre-analytical workflow and extraction.
5	Pyrosequencing Analysis of Cryogenically Ground Samples from Primary and Secondary/Persistent Endodontic Infections	10.1016/j.joen.2017.03.019	Include	Provides a valuable workflow using cryogenic grinding, DNA extraction, and pyrosequencing in human endodontic samples.
6	A cross-sectional metagenomic analysis of the microbial ecology in symptomatic apical periodontitis-An in vivo study	10.4103/JCDE.JCDE_963_25	Include	In vivo human endodontic study using NGS with sufficient methodological detail.
7	Amplicon-based next-generation sequencing for comparative analysis of root canal microbiome of teeth with primary and persistent/secondary endodontic infections	10.1007/s00784-023-04882-x	Include	Clearly eligible: human endodontic context, NGS, and adequate methodological reporting.
8	Microbial Communities in the Extraradicular and Intraradicular Infections Associated With Persistent Apical Periodontitis	10.3389/fcimb.2021.798367	Include	Human endodontic and periapical samples with NGS and reported extraction workflow.
9	Distinctive microbiota distribution from healthy oral to post-treatment apical periodontitis	10.3389/fcimb.2022.980157	Include	Includes human periapical samples related to endodontic infection and uses NGS.
10	Analysis of the diversity and function of the microbiota in infected root canals of type 2 diabetes mellitus with apical periodontitis	10.3389/fcimb.2025.1514510	Include	Human endodontic infection study with sequencing and sufficient methodological utility for the review.
11	Quantification of Bacterial Colonization in Dental Hard Tissues Using Optimized Molecular Biological Methods	10.3389/fgene.2020.599137	Include	Optimizes DNA isolation in human teeth with explicit applicability to NGS workflows.
12	Root microbiota in primary and secondary apical periodontitis	10.3389/fmicb.2018.02374	Include	Human study using MiSeq with informative sampling and DNA extraction methods.
13	Microbiome in paired root apices and periapical lesions and its association with clinical signs in persistent apical periodontitis using next-generation sequencing	10.1111/iej.13893	Include	Methodologically strong; paired human samples with cryopulverization and MiSeq.
14	Impact of root canal disinfection on the bacteriome present in primary endodontic infection: A next generation sequencing study	10.1111/iej.14074	Include	Clinical human study with NGS and adequate detail on pre-analytical workflow.
15	Identification of keystone taxa in root canals and periapical lesions of post-treatment endodontic infections: Next generation microbiome research	10.1111/iej.14046	Include	Eligible by context and NGS; note possible partial cohort overlap.
16	Illumina MiSeq Sequencing for Preliminary Analysis of Microbiome Causing Primary Endodontic Infections in Egypt	10.1155/2018/2837328	Include	Reports disinfection, DNA extraction, negative control, and MiSeq sequencing.
17	Pyrosequencing Analysis of the Apical Root Canal Microbiota	10.1016/j.joen.2011.08.012	Include	Classic eligible study using cryogenic grinding, DNA extraction, and pyrosequencing.
18	Bacterial flora of dental periradicular lesions analyzed by the 454-pyrosequencing technology	10.1016/j.joen.2012.06.037	Include	Human endodontic periradicular lesions; eligible under the PCC and uses NGS.
19	Effects of Sequential Antimicrobial Phases on Root Canal Microbiome Dynamics in Two-Visit Treatment of Primary Apical Periodontitis: A	10.3390/life14121696	Include	Uses MiSeq in human clinical samples across multiple treatment phases.

No.	Title	DOI	Final decision	Rationale
	Longitudinal Experimental Study			
20	Microbial Analysis of Endodontic Infections in Root-filled Teeth with Apical Periodontitis before and after Irrigation Using Pyrosequencing	10.1016/j.joen.2017.11.019	**Include**	Human study with NGS, contamination controls, and adequately reported extraction.
21	Characterization of Root Canal Microbiota in Teeth Diagnosed with Irreversible Pulpitis	10.1016/j.joen.2020.12.009	**Include**	Human pulpal/endodontic context with MiSeq and relevance to the methodological question.
22	Residual Bacteriome after Chemomechanical Preparation of Root Canals in Primary and Secondary Infections	10.1016/j.joen.2022.03.008	**Include**	Human NGS study assessing samples before and after chemomechanical preparation.
23	Impact of an Enhanced Infection Control Protocol on the Microbial Community Profile in Molar Root Canal Treatment: An in Vivo NGS Molecular Study	10.1016/j.joen.2022.08.008	**Include**	Highly useful for the review; includes controls, extraction details, and MiSeq.
24	Clinical Investigation of Bacteriome in Primary Endodontic Infections With Apical Periodontitis Using High-Throughput Sequencing Analysis	10.1016/j.joen.2024.07.016	**Include**	Methodologically strong study with DNA/RNA Shield, extraction workflow, and MiSeq.
25	Microbiome in the apical root canal system of teeth with post-treatment apical periodontitis	10.1371/journal.pone.0162887	**Include**	Provides apical sampling, cryopulverization, DNA extraction, and MiSeq sequencing.
26	Pyrosequencing analysis of apical microbiota of teeth with persistent apical periodontitis	10.1016/j.jds.2015.06.001	**Include**	Eligible human study with cryogenic grinding and pyrosequencing.
27	Impact of root canal preparation using two single-file systems on the intra-radicular microbiome of teeth with primary apical periodontitis	10.1007/s00784-024-05544-2	**Include**	Includes serial sampling, extraction, MiSeq, and contaminant removal.
28	Microbiota in the apical root canal system of tooth with apical periodontitis	10.1186/s12864-019-5474-y	**Include**	Contains a clearly relevant apical periodontitis arm with MiSeq and reported extraction.
29	Profiling of microbes associated with chronic irreversible pulpitis using metagenomic next-generation sequencing	10.1186/s12903-025-07175-9	**Include**	Excellent fit with the PCC; shotgun metagenomics and well-described workflow.
30	Association between root canals and gingival sulci microbiota in secondary and persistent endodontic infections	10.1038/s41598-025-95522-9	**Include**	Root canal samples are central, with extraction details, controls, and MiSeq.

Excluded reports (n = 14)

No.	Title	DOI	Final decision	Rationale
1	Presence of archaea and selected bacteria in infected root canal systems	10.1139/cjm-2017-0531	**Exclude**	No NGS; used PCR/sqRT-PCR and direct sequencing.
2	Nonsurgical Retreatment of a 16 × 9 mm Osteolytic Lesion With Buccal Bone Regeneration Using a Bioceramic Sealer: A Clinical and NGS-Based Microbiological Case Report With 30-Month Follow-Up	10.1155/crid/9703223	**Exclude**	Insufficient methodological reporting to map sampling, extraction, controls, and sequencing workflow.
3	Evaluation of sodium hypochlorite irrigant, bingpeng irrigant, and fufang bingpeng irrigant as endodontic irrigants during passive ultrasonic irrigation	10.3389/fcimb.2019.00145	**Exclude**	Insufficient reporting of the DNA extraction method for the review objective.
4	The impact of an enhanced infection control protocol on molar root canal treatment outcome – a randomised clinical trial	10.1111/iej.13605	**Exclude**	No NGS; based on qPCR.
5	Microflora profiling of infected root canal before and after treatment using culture-independent methods	10.1007/s12275-012-0459-4	**Exclude**	No NGS; used PCR/cloning/partial sequencing.
6	Culture-independent Characterization of the Microbiome of Healthy Pulp	10.1016/j.joen.2018.03.009	**Exclude**	Population outside the PCC; healthy pulp rather than infected endodontic tissue.
7	Molecular profiling of antibiotic resistance genes in acute and chronic irreversible pulpitis: A cross-sectional study	10.4103/JCDE.JCDE_311_25	**Exclude**	No NGS and primary focus on antimicrobial resistance genes.
8	Patterns of oral microbiota in patients with apical periodontitis	10.3390/jcm10122707	**Exclude**	No NGS; used culture and real-time PCR.
9	The influence of apical periodontitis on the concentration of inflammatory mediators in peripheral blood plasma and the metagenomic profiling of endodontic infections: Study design and protocol	10.1016/j.conctc.2020.100686	**Exclude**	Protocol paper without primary study results.
10	The Apical Root Canal System of Teeth with Posttreatment Apical Periodontitis: Correlating Microbiologic, Tomographic, and Histopathologic Findings	10.1016/j.joen.2020.05.020	**Exclude**	No NGS; used real-time PCR.
11	Assessing the Iatrogenic Contribution to Contamination During Root Canal Treatment	10.1016/j.joen.2022.01.007	**Exclude**	NGS was used, but the study focused on operator-related contamination sites rather than core endodontic clinical samples or validation of endodontic DNA extraction workflows.
12	Controlling Root Canal Infection in Oncological Patients with Apical Periodontitis	10.1016/j.joen.2024.11.012	**Exclude**	No NGS; used qPCR.
13	Relationship of MMP-9 with the clinical course of apical periodontitis and the main bacterial species in the oral microbiota	10.1007/s10266-023-00895-w	**Exclude**	No NGS; used PCR and Sanger sequencing.
14	Characterization of microbiota of root canal-treated teeth with posttreatment disease	10.1128/JCM.00531-12	**Exclude**	No NGS; used DNA-DNA hybridization and qPCR.
